# Bridging the gap: assessing CMV DNAemia in kidney transplant recipients with previous solid organ transplants

**DOI:** 10.3389/frtra.2024.1280280

**Published:** 2024-04-09

**Authors:** Goni Katz-Greenberg, Julie M. Steinbrink, Krishna Shah, Jennifer S. Byrns

**Affiliations:** ^1^ Division of Nephrology, Department of Medicine, Duke University School of Medicine, Durham, NC, United States; ^2^ Division of Infectious Disease, Department of Medicine, Duke University School of Medicine, Durham, NC, United States; ^3^ Department of Pharmacy, Duke University Hospital, Durham, NC, United States

**Keywords:** CMV, solid organ transplant, kidney transplantation, immunosuppression, prophylaxis

## Abstract

Cytomegalovirus (CMV) infection poses a significant threat to solid organ transplant (SOT) recipients and can lead to various complications and adverse outcomes. In an effort to prevent CMV infection, it is common to utilize prophylactic strategies, including antiviral medications such as valganciclovir, especially for high-risk patients. Risk factors for CMV infection in kidney transplant recipients (KTRs) include CMV mismatch between donor and recipient (i.e., donor positive, recipient negative), and intensity of immunosuppression, such as the use of T-cell depleting agents. However, little attention has been given to KTRs with a history of prior SOTs, despite their prolonged exposure to immunosuppressive regimens. The aim of this retrospective single-center study was to investigate the incidence and implications of CMV DNAemia in KTRs with prior SOTs. The study included 97 KTRs with prior SOTs and 154 KTRs with no prior transplants as a control group. In the study group, the most common SOT before the current kidney transplantation (KT), was a previous KT. Patients in the KTR group with prior SOTs were more sensitized than those in the control group [calculated panel-reactive antibody > 30%: 49 (50.5%) vs. 30 (19.45%) patients, *p* = 0.001]. There was a 39.2% incidence of CMV DNAemia in the previous SOT group compared to 48.7% in the control group [non-significant (NS)]. Patients with prior SOTs demonstrated a shorter post-transplant time to CMV DNAemia [median time 1.6 months (interquartile range, IQR 0.7–5.8) in the KTRs with prior SOTs vs. 2.6 months (IQR 1.5–8.1) in the control group (*p* = 0.001)]. Although the study highlights the need for tailored prophylaxis strategies and vigilant monitoring in KTRs with prior SOTs, its limitations, such as its retrospective nature and single-center design, call for further multicenter research to establish comprehensive guidelines for managing CMV DNAemia in this unique patient population. Despite these limitations, this study underscores the importance of recognizing the heightened risk of CMV infection or reactivation in KTRs overall and the potential benefits of proactive intervention to mitigate associated morbidity and mortality.

## Introduction

Cytomegalovirus (CMV) remains one of the most significant infections impacting solid organ transplant (SOT) recipients (1, 2). Beyond the immediate comorbidity associated with CMV DNAemia, the immune-modulating impacts of CMV make patients more susceptible to various complications, including increased rates of other opportunistic infections, graft loss, and even death (1, 3, 4).

A pivotal strategy in evaluating CMV risk revolves around the pre-transplant categorization of patients based on their CMV serostatus, which ultimately shapes the choice and duration of antiviral prophylaxis and may vary across different transplant centers (5, 6). Some centers opt for pre-emptive therapy, a vigilant approach of monitoring, and treating CMV reactivation if it occurs, while others adhere to the primary prophylaxis, aiming to prevent initial CMV activation altogether (6, 7).

However, the scenario becomes more intricate when considering kidney transplant recipients (KTRs) who have undergone prior SOTs. This subgroup of recipients presents a unique challenge due to their prolonged exposure to immunosuppressive regimens, possible heightened sensitization profiles, and the need for lymphocyte depletion, stemming from their history of previous transplants. Yet, the majority of CMV risk assessment algorithms do not include a history of previous SOTs as a factor in determining the susceptibility to CMV DNAemia (3).

The aim of the present study was to examine the incidence and timing of CMV DNAemia as well as its effects on patient and graft survival among KTRs who have undergone prior SOTs at our transplant center.

## Material and methods

### Study design

This was a retrospective, single-center study conducted to evaluate the occurrence of CMV DNAemia in patients who received a kidney transplant (KT) between 1 January 2014 and 1 May 2021 with a history of prior SOT, and who met 1 year of follow-up after transplant. Patients with primary non-function were excluded from the analysis. A control group of KT patients transplanted during the same period at our institution was used for the outcome comparison. In the control group, previous SOTs of any kind were excluded.

This study was approved by the Institutional Review Board (IRB) at Duke University Medical Center (Pro00110030) and was performed in accordance with the Declaration of Helsinki. Data were collected and stored securely within our institutions’ REDCap database.

### Maintenance immunosuppression

The induction of immunosuppressive therapy was determined per our center's protocol based on panel-reactive antibodies (PRAs) for human leukocyte antigen (HLA) class I and II, with methylprednisolone for PRAs <30%, and antithymocyte globulin (ATG) for patients with PRAs ≥30%. Patients at high risk for delayed graft function (DGF) also received ATG per the team’s discretion. The initial maintenance immunosuppression included a calcineurin inhibitor, mycophenolate mofetil (MMF), and a corticosteroid.

### CMV prophylaxis protocol

Per our institutional protocol, all CMV high-risk patients (seropositive donor/seronegative recipient, D+/R−) and seropositive recipients from seropositive donors (D+/R+) who received ATG for induction received universal prophylaxis with oral valganciclovir for a period of 6 months. All other transplant recipients underwent a CMV pre-emptive therapy approach (i.e., no antiviral initiation unless CMV was detected on closely monitored surveillance). All transplant recipients underwent routine CMV polymerase chain reaction (PCR) monitoring every 2 weeks for the first 3 months and then monthly for the remainder of the first post-transplant year.

### Objectives

The primary endpoint was the incidence of CMV DNAemia at 1 year. The secondary endpoints included time to CMV DNAemia after transplant, allograft function defined by serum creatinine and glomerular filtration rate (GFR) at 1 year, patient and graft survival rates, and frequency of biopsy-proven acute rejection (BPAR).

### Statistical analysis

The primary outcome, incidence of CMV DNAemia, was a categorical variable that was analyzed using a chi-square test. The secondary outcome, time to CMV DNAemia, was a non-normally distributed continuous variable analyzed using the Mann–Whitney *U* test. Allograft function defined as serum creatinine and GFR were also non-normally distributed continuous variables analyzed using the Mann–Whitney *U* test. The incidence of BPAR, a categorical variable, was analyzed using Fisher's exact test.

Baseline demographics were expressed as absolute numbers and percentages for categorical data and as medians with interquartile ranges (IQR) or means with standard deviations (SD) for skewed distribution. Baseline demographics between groups were compared using *t*-tests for normally distributed data, the Mann–Whitney *U* test for non-normally distributed data, and the chi-square or Fisher's exact tests for categorical data based on the sample size using JMP® Pro 17.20.0 and GraphPad Prism® version 10.0.0. A power analysis was not calculated given the limited number of patients in this retrospective cohort analysis.

## Results

During the study period, 97 patients met the inclusion criteria for the previous SOT group and 154 received their first KT as a control. The majority of patients were male (61.9% vs. 63.6% in the previous SOT group vs. control group, respectively) and white (53.6% vs. 47.4%) (Table 1). The median time between prior SOT to most recent KT was 152.19 months (IQR 85.82–271.43). For almost two-thirds of patients (63.9%), the original organ transplanted was also a kidney.

**Table 1:** Baseline demographics.

	KT following SOT (*n* = 97)	Control (*n* = 154)	*p*-value
Age years, mean (SD)	50.5 (13.4)	55.9 (11.2)	0.002
Female, *n* (%)	37 (38.1)	56 (36.4)	0.26
Race, *n* (%)			
Black	37 (38.1)	72 (46.8)	0.16
White	52 (53.6)	73 (47.4)	0.28
Other	8 (8.2)	9 (5.8)	0.29
Previous transplant type, *n* (%)		N/A	N/A
Kidney	62 (63.9)		
Heart	15 (15.5)		
Liver	7 (7.2)		
Lung	6 (6.2)		
Pancreas	1 (1)		
Kidney/pancreas	4 (4.1)		
Liver/lung	2 (2.1)		
Time from last SOT to current KT (months), median (IQR)	152.19 (85.82–271.43)	N/A	N/A
Kidney donor type, *n* (%)			0.29
Living donor	29 (29.9)	56 (36.4)	
Deceased donor	68 (70.1)	98 (63.6)	
cPRA >30%, *n* (%)	49 (50.5)	30 (19.4)	0.001
Cold ischemic time (hours), median (IQR)	17.5 (2.1–24.0)	15.4 (1.9–23.3)	0.79
KDPI, median (IQR)	38.5 (24.3–57.5)	49.5 (36–75)	0.0002
Delayed graft function, *n* (%)	22 (22.7)	41 (26.6)	0.55
CMV serostatus, *n* (%)			
CMV D+/R−	20 (20.6)	36 (23.4)	0.84
CMV D+/R+	40 (41.2)	60 (39.0)	0.72
CMV D−/R+	24 (24.7)	22 (14.3)	0.04
CMV D−/R−	13 (13.4)	36 (23.4)	0.05

cPRA, calculated panel-reactive antibody; KDPI, kidney donor profile index.

Patients in the previous SOT group were considered at higher immunologic risk, with a mean PRA of 32% class I and 30% class II. More than 80% of patients in the control group had a PRA <30%, putting them at lower immunologic risk (Table 1). In the KT following SOT cohort, 55.7% of patients received T-cell depleting agents (antithymocyte globulin or alemtuzumab) vs. 50.9% of the control (*p* = 0.56).

More than 60% of recipients were CMV serostatus positive (D+/R+ and D−/R+) at the time of KT in the previous SOT group compared to 53.3% in the control group. Of them, 20.6% of patients in the previous SOT group and 23.0% of patients in the control group were classified as high risk for CMV (D+/R-) (Table 1). For CMV prophylaxis, 63.9% of patients received valganciclovir initiated immediately after transplant in the KT following SOT group compared to 55.8% of patients in the control group (Table 2).

**Table 2:** Overall immunosuppression, CMV prophylaxis.

	KT following SOT (*n* = 97)	Control (*n* = 154)	*p*-value
Induction, *n* (%)			0.56
T-cell depletion (antithymocyte globulin and alemtuzumab)	54 (55.7)	80 (50.9)	—
Basiliximab	16 (16.5)	1 (0.6)	0.002
Methylprednisolone	27 (27.8)	73 (47.4)	
Maintenance immunosuppression, *n* (%)			
Tacrolimus/mycophenolate/prednisone	80 (82.5)	141 (91.6)	0.04
Cyclosporine/mycophenolate/prednisone	3 (3.1)	0	—
Belatacept	3 (3.1)	12 (7.8)	0.12
Other	12 (12.4)	1 (0.6)	—
CMV prophylaxis, *n* (%)			
Valganciclovir	62 (63.9)	86 (55.8)	0.21
Acyclovir or valacyclovir	31 (32.0)	63 (40.9)	0.15
None	4 (4.1)	5 (3.2)	0.72

For the primary outcome, the overall incidence of CMV DNAemia was found to be 39.2% in the previous SOT group and 48.7% in the control group, which was not statistically significant between groups (*p* = 0.14) (Table 3). Of the patients who developed CMV DNAemia in the previous SOT group, 10 (26.3%) patients had quantitative CMV PCRs of a high enough threshold to warrant treatment with oral valganciclovir or intravenous ganciclovir. In the control group, 38 (50.7%) patients required CMV treatment. The remainder had low-level DNAemia that required solely additional monitoring and immunosuppression reduction, based on our transplant center's protocol.

**Table 3:** Outcomes.

	KT following SOT (*n* = 97)	Control (*n* = 154)	*p*-value
CMV DNAemia incidence, *n* (%)	38 (39.2)	75 (48.7)	0.14
Time to CMV DNAemia post-KT (months), median (IQR)	1.6 (0.7–5.8)	2.6 (1.5–8.1)	0.04
Serum creatinine (mg/dl) at 1 year, median (IQR)	1.4 (1.1–1.7)	1.4 (1.1–1.7)	0.70
GFR at 1 year, median (IQR)	54 (43–64)	52 (43–63.8)	0.83
BPAR, *n* (%)			
TCMR (grade 1a or higher)	3 (3.1)	8 (5.2)	0.43
ABMR	2 (2.1)	1 (0.6)	—

TCMR, T-cell-mediated rejection; ABMR, antibody-mediated rejection.

For secondary outcomes, the median time from transplant to CMV DNAemia was 1.6 months (IQR 0.7–5.8) in the previous SOT group and 2.6 months (IQR 1.5–8.1) in the control group, which was statistically significant (*p* = 0.001) (Table 3). Serum creatinine was not different between the groups at 1 year [1.4 mg/dl (IQR 1.1–1.7) in both groups, *p* = 0.23]. GFR at 1 year was also not different between the groups [54 ml/min (IQR 43–64) vs. 52 ml/min (IQR 43–63.8), *p* = 0.76]. For-cause biopsies were performed in 16 patients, and BPAR was noted in 3 (3.1%) patients in the KT following SOT group vs. 8 (5.2%) patients in the control group (*p* = 0.43). Antibody-mediated rejection was noted in two (2.1%) patients in the KT following SOT group vs. one (0.6%) patient in the control group. Patient survival was 100% in both cohorts. One patient lost their kidney allograft due to BK nephropathy during the 1-year follow-up in the KT following SOT group.

## Discussion

Despite screening, effective antiviral drugs, and risk-balanced prophylaxis, CMV remains a major cause of morbidity and mortality in KTRs (1, 8). This risk may be elevated in patients with previous transplants given the extended exposure to immunosuppression and the resulting myelosuppression. Anecdotal evidence from caring for these patients has indicated a potentially higher incidence of CMV DNAemia, warranting the need for this study. In our cohort, when assessing the incidence of CMV DNAemia in those patients with previous transplants undergoing subsequent KT vs. those receiving their first KT, there was no significant difference between the groups. Despite the prior SOT group being more sensitized (Table 1), both groups were well balanced in the degree of immunosuppression based on the use of T-cell depleting agents (Table 2). In addition, there was no difference in CMV high-risk serostatus between the groups (Table 1).

In the literature, the characteristic of a previous transplant is not included in risk factor analyses. Parameters such as sensitization and lymphocyte depletion requirements, which may describe the previous transplant patient, are included (9). This does not account for the degree of bone marrow suppression over time that patients may have endured. Leukopenia, which helps describe this potential bone marrow suppression, is more often seen in previously transplanted patients (10). In our cohort, almost two-thirds of patients in the previous SOT group developed leukopenia within the first year after transplant. Despite more cases of leukopenia, there was no difference in rate of CMV DNAemia in this study. More than 50% of the previous SOT cohort did receive lymphocyte depletion and, per our protocol, CMV prophylaxis with valganciclovir was given to these patients if they were at intermediate to high risk for CMV reactivation (CMV D+/R−, or R+), which may explain why they did not develop CMV DNAemia. Our institution also minimizes immunosuppression when leukopenia occurs, which helps decrease the risk for viral reactivation as well.

While KTRs are more susceptible to acquiring or reactivating CMV during the first 100 days after transplant (11, 12), it is important to note that CMV DNAemia and CMV disease can occur at any point after transplantation (13). In our cohort, patients with a previous SOT had a shorter time to viremia than those in the control group (1.6 vs. 2.6 months). There are a few important caveats that can affect the time to viremia, such as the use of CMV prophylaxis vs. pre-emptive monitoring as well as further investigation of the appropriate dosing of CMV prophylaxis in the setting of changing kidney function after KT.

A total of 38 patients who had CMV DNAemia also had BK reactivation in the study period (13 in the previous SOT group and 25 in the control group). Co-infection with BK virus and CMV in KTRs is significantly associated with decreased allograft function. Since co-infection is strongly associated with acute rejection, co-infected individuals should be considered a high-risk collective (14).

The present study has some limitations. First, its retrospective design introduces inherent biases and limitations in data collection. The limited number of patients in the study did not allow for it to be powered to find a statistically significant difference between the groups. In addition, we did not collect data regarding recipients’ comorbidities and did not have data available regarding CMV serostatus and history of CMV infection during the prior transplant. Lastly, the single-center nature of the study limits the generalizability of the results to broader populations. Future research endeavors should focus on conducting large-scale, multicenter studies to validate these findings and establish more comprehensive guidelines for managing CMV DNAemia in KTRs with prior SOTs.

In conclusion, we believe previous exposure to immunosuppressive treatment, here in the setting of prior SOTs, may play a factor in CMV reactivation risk and/or viremia given the degree of myelosuppression these patients may have coming into and after an additional transplant. CMV prophylaxis determinations are important for these patients. Further data are needed to establish more precise guidelines and recommendations.

## Data Availability

The raw data supporting the conclusions of this article will be made available by the authors, without undue reservation.
